# Eco-Friendly Extraction of Sustainable and Valorized Value-Added Products From *Ulva fasciata* Macroalgae: A Holistic Technoeconomic Analysis

**DOI:** 10.1155/ijbm/5811057

**Published:** 2025-02-26

**Authors:** Nour Sh. El-Gendy, M. Shaaban Sadek, Hussein N. Nassar, Ahmad Mustafa

**Affiliations:** ^1^Process Design and Development Department, Egyptian Petroleum Research Institute (EPRI), P.O. Box 11727, Nasr City, Cairo, Egypt; ^2^Center of Excellence, October University for Modern Sciences and Arts (MSA), P.O. Box 12566, 6th of October City, Giza, Egypt; ^3^Chemical Engineering Department, Faculty of Engineering, Minia University, P.O. Box 61519, Minya, Egypt; ^4^Biochemistry Program, Faculty of Physical Therapy, October University for Modern Sciences and Arts (MSA), P.O. Box 12566, 6th of October City, Giza, Egypt; ^5^General Systems Engineering Department, Faculty of Engineering, October University for Modern Sciences and Arts (MSA), 6th of October City, Egypt

**Keywords:** biorefinery process, internal rate of return, payback period, return on investment, simulation, *Ulva fasciata*

## Abstract

The present work conducts a detailed technoeconomic analysis of an environmentally friendly zero-waste biorefinery process to valorize marine *Ulva fasciata* macroalgae into different sustainable value-added products. The proposed sequential fully integrated process yielded 34.89% mineral-rich water extract (MRWE), 2.61 ± 0.5% chlorophyll, 0.41 ± 0.05% carotenoids, 12.55 ± 1.6% starch, 3.27 ± 0.7% lipids, 22.24 ± 1.8% ulvan, 13.37 ± 1.5% proteins, and 10.66 ± 0.9% cellulose. The Aspen Plus software, utilizing the nonrandom two-liquid (NRTL) model, was applied for process design, simulation, and technoeconomic analysis. Key findings include a positive net present value (NPV) of $49,755,544.90, a high return on investment (ROI) of 485%, and an internal rate of return (IRR) of 17%. The anticipated payback period is 7 years, indicating a quick recovery of the initial investment. These findings confirm that *Ulva fasciata* is a promising resource in the biorefinery industry, providing a viable and eco-friendly alternative for the production of bio-based products and a new market for seaweed-based products.

## 1. Introduction

Environmental conservation and economic development increasingly recognize the global significance of sustainable bioresource utilization [1]. Marine macroalgal biomass, particularly species like *Ulva fasciata*, plays a crucial role in this paradigm. *Ulva fasciata*, a type of green seaweed, is abundant in the Mediterranean shoreline, fast-growing, and rich in valuable compounds [2]. The proliferation of such green macroalgae often results in eutrophication in enclosed coastal areas and adversely affects the marine ecosystem, aquaculture, touristic activities, and economy [3, 4]. Its application in green chemistry and biorefinery represents a sustainable approach to resource management and presents a viable strategy for ecosystem bioremediation, efficient use of marine macroalgal resources, and development of a green and low-carbon economy. Reliance on fossil fuels can be reduced and associated environmental impacts mitigated, by converting marine biomass into a range of value-added products, such as biofuels, pharmaceuticals, and biodegradable polymers [5]. Moreover, this approach aligns with circular and blue economy principles, promoting resource efficiency and waste reduction while fostering innovation in green technology [3].

Using the biorefinery approach on *Ulva fasciata* involves a unified process for valorizing this marine biomass into a wide range of sustainable and valuable products [3, 6]. This approach is grounded in the principles of sustainability, efficiency, and value maximization. It aims to extract maximum value from raw materials while minimizing waste. Consequently, all components of *Ulva fasciata* biomass are used to produce valuable products, including biofuels, pharmaceuticals, nutraceuticals, and biodegradable polymers [7]. The potential of this approach lies in its ability to enhance resource efficiency and diversify products, aligning with both environmental sustainability and economic viability [8]. By implementing integrated processes, every part of *Ulva fasciata* is valorized, supporting a more sustainable and circular bioeconomy.

Given the limited feasibility studies on macroalgae-based biorefinery processes [9, 10], a technoeconomic analysis (TEA) is essential to assess their feasibility and viability. TEA provides a comprehensive assessment of both technical aspects (such as yield, efficiency, and scalability) and economic factors (such as revenue potential and return on investment [ROI]) [11]. TEA is crucial for identifying economic barriers and technical obstacles in macroalgal biorefinery. This information can guide research and development toward more sustainable and practical solutions. Moreover, it aids in investment decision-making and policy development, ensuring the sustainable and profitable use of macroalgae as a bioresource [12].

The conversion of the problematic bloomed green macroalgae into various sustainable valued products and biopolymers has recently garnered significant interest due to its extensive applications in the food, animal feed, pharmaceutical, cosmetic, dye, organic fertilizer, biofuel, and biodegradable plastic industries [3, 4]. In earlier documented fully integrated routes, Indian *Ulva fasciata* biomass was converted into five valued products, including a mineral-rich liquid extract, lipids, ulvan, cellulose, and bioethanol [4]; *Ulva lactuca* was transformed into five valued products, including mineral-rich sap, lipids, ulvan, proteins, and cellulose [13]; and *Ulva ohnoi* was processed into six valued products, including salts, starch, lipids, ulvan, proteins, and cellulose [9]. As far as we know, there is no documented completely integrated zero-waste eco-friendly practice for the conversion of *Ulva fasciata* into eight valued products, including chlorophyll_a,b_, carotenoids, mineral-rich water extract (MRWE), lipids, and proteins, in addition to the valuable biopolymers: starch, ulvan, and cellulose. Thus, in our previous work [3], we experimentally reported a fully integrated biorefinery process for the eco-friendly valorization of *Ulva fasciata* into various green and sustainable value-added products. The findings from that piece of paper prompted us to simulate the process to gain a deeper understanding of the process economy. Consequently, this work aims to present the pioneering complete TEA for valorizing *Ulva fasciata* into various valued products using the previously published zero-waste biorefinery approach [3]. This was performed by simulating the whole bioprocess using the Aspen Plus software and then figuring out the economy indicators, such as the payback period, positive net present value (NPV), ROI, and internal rate of return (IRR).

## 2. Material and Methods

### 2.1. Process Description

**Figure 1:**
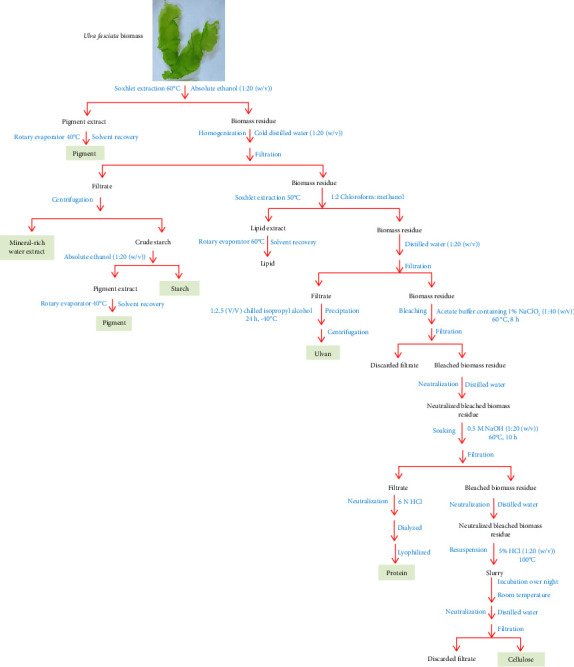
A fully integrated process for the valorization of *Ulva fasciata* into sustainable value-added products [3].

A fully integrated process for the extraction of value-added products from the collected *Ulva fasciata* was previously reported [3].  Figure 1 presents the schematic diagram for the previously published experimental work [3]. The Aspen Plus software (V.10, Aspen Tech Inc., Bedford, Massachusetts, USA) was employed to model the extraction of value-added products derived from *Ulva fasciata* macroalgae. The activity coefficient nonrandom two-liquid (NRTL) thermodynamic model, introduced by Renon and Prausnitz in 1968 [14], was used as the property method to simulate this process. This model is specifically recommended for processes involving valorizing seaweed biomass into proteins, lipids, carbohydrates, and similar substances. The composition of the biomass, detailed in Table 1 and obtained from a prior study [3], was adopted for the simulation. The Aspen Plus databases align most components, while the National Renewable Energy Laboratory (NREL) developed a physical property database to define any unavailable elements [15, 16].

The short-cut distillation model (DSTWU) was applied in the modeling process to forecast the production of pigments and lipids, while the Radfrac model facilitated the separation of isopropyl alcohol via azeotropic distillation. Given the significant difference in boiling points between water and ethylene glycol, a distillation column was applied to recover the solvent for future reuse. This approach was implemented across all distillation towers used throughout the process. To maintain the tank's temperature, a mixer equipped with a heater was utilized. In addition, simulations involving membranes and filters were executed using RStoic and component separator tools, respectively, leveraging known conversion percentages. The heater performance metrics and properties across named units are illustrated in supporting information Table S1, while all data related to process heat exchangers are illustrated in supporting information Table S2. The DSTWU performance and key component recovery across designated units are illustrated in supporting information Table S3. The RStoic analysis across designated units is illustrated in supporting information Table S4. The operating conditions were taken from our previous work [3].

### 2.2. Economic Analysis

The main goals of economic studies include identifying revenue sources, evaluating project management and implementation expenses, and assessing project feasibility through financial analysis. This ongoing technoeconomic research offers a thorough overview that can assist decision-making regarding value-added products derived from *Ulva fasciata.*

#### 2.2.1. Synopsis of Overall Capital Investment

The examination delves into the significance of precisely determining the total capital investment for this hypothetical project. This involves aggregating fixed capital investment, working capital, and initial expenditure [17]. Given the long-term implications for the plant's planning, it becomes critical to consider the overall spending investment [11, 18]. The necessity to avoid impractical budgets underscores the crucial role of accurate costing. This encompasses engineering costs, both inside battery limits (ISBLs) and outside battery limits (OSBLs), as well as contingency expenses for diverse production-related accessories and equipment. Estimation techniques developed by Bridgewater, Taylor, Gore, Stallworthy, Klumpar, Brown, and Fromme can calculate the ISBL cost [19]. In this instance, Bridgewater's approach was utilized. The accuracy of Bridgewater's methodologies depends on specific factors such as the reactor's conversion rate, plant capacity, and the number of main units. The study determined the plant's capacity to be 7920 tonnes per year. Finally, equation (1) within Bridgewater's method was utilized to compute the ISBL cost for this scenario [18]:

(1) $$\begin{array}{c} C=280,000\;N{\left(\frac{Q}{S}\right)}^{0.3}, \end{array}$$

where *C* denotes the ISBL capital cost (in £), *N* signifies the number of main units, *Q* represents the plant capacity in tons per year, and *s* stands for the reactor conversion rate.

The OSBL cost covers off-site development for plant operations, typically falling between 10% and 100% [20]. However, this work applied a 30% OSBL ratio. The company's research team possessed the necessary package, which excluded engineering fees. The project earmarked a 10% contingency to accommodate unforeseen expenses. 15% of the direct capital cost was designated as working capital [18, 21]. Start-up expenses accounted for 15% of the combined OSBL and ISBL costs.  Table 2 details other economic assumptions utilized in this study.

#### 2.2.2. Operating Costs

Operating costs include both fixed and variable production expenses. Irrespective of a project's efficiency, fixed costs such as labor, overhead, maintenance, insurance, taxes, rent, and environmental fees are inherent [22]. Conversely, variable costs fluctuate with production rates and outputs. These include expenses for raw materials such as biomass, ethanol, methanol, chloroform, isopropyl alcohol, sodium hypochlorite, acetic acid, sodium acetate, HCl, NaOH, and ethylene glycol, as well as utilities such as energy, heating and cooling water, transportation, packaging, and waste disposal. Overall, effective resource utilization, such as reducing raw material losses and energy consumption, can help to reduce variable costs [18]. Raw materials, being notably costly, contribute significantly to the overall production expenses.

#### 2.2.3. Economic Viability Indicators

The investment's income comes from selling its products, including both the primary output and any secondary products. Calculating the gross margin involves deducting the raw material costs from the revenue generated by product sales. This margin indicates that retained revenues exceed production expenses. In this study, to determine profit, the cash cost of production (CCOP) was deducted from the product revenues, yielding the gross profit. Subsequently, the net profit was computed by subtracting the corporate tax rate, which was established in this study as 22.5%, from the gross profit using equation (2) to find the tax amount:

(2) $$\begin{array}{c} \text{tax paid}=\text{taxable income}-\text{tax rate}, \end{array}$$

(3) $$\begin{array}{c} \text{taxable income}=\text{total income}-\text{deductions}-\text{exemptions}. \end{array}$$

Equation (4) calculates the simple payback period by dividing the fixed investment by the average annual cash flow, taking into account revenue-generating years from 3 through 15:

(4) $$\begin{array}{c} \text{payback time}=\frac{\text{total investment}}{\text{average annual cash flow}}. \end{array}$$

The depreciation expenses are calculated using the declining balance depreciation method, particularly in scenarios that prioritize cash flow. This study took into account a 10% depreciation rate over a 15-year project lifespan, including a 5-year project recovery phase. The ROI is ascertained by subtracting expenses from income, resulting in the net profit [23]. However, this study suggested an 11% discount rate. The economic metric, called NPV, estimates the disparity between the current values of cash inflows and outflows. The annualization of an interest rate was employed to consider the time value of money. Equation (5) was utilized to calculate the NPV [18]:

(5) $$\begin{array}{c} \text{NPV}=\overset{n=t}{\underset{n=1}{\sum }}\frac{{\text{CF}}_{n}}{{\left(1+i\right)}^{n}}, \end{array}$$

where the CF_*n*_ represents the cash flow occurring in each specific year, *i* denotes the discount rate, and *t* signifies the duration of the project in years.

ROI serves as a ratio that evaluates the profitability of an investment. A higher ROI percentage suggests that the returns from the investment are anticipated to surpass the incurred expenses. The calculation of ROI is determined by equation (6) [24]:

(6) $$\begin{array}{c} \text{ROI}=\left(\frac{\text{cumulative net profit}}{\text{initial investment}\times \text{plant life}}\right)\times 100. \end{array}$$

Regarding the IRR, it can be computed using equation (7) or by using the IRR function in MS Excel:

(7) $$\begin{array}{c} \text{IRR}=\overset{n=t}{\underset{n=1}{\sum }}\frac{{\text{CF}}_{n}}{{\left(1+i^{\prime }\right)}^{n}}=0, \end{array}$$

where CF_*n*_ represents the cash flow in year *n*, *t* denotes the project life in years, and *i*′ stands for the discounted cash flow rate of return (expressed as a fraction).

## 3. Results and Discussion

### 3.1. The Yields of the Produced Value-Added Products

The proposed zero-waste biomass residue (BIOMSRES) technique yielded 34.89% MRWE, 2.61 ± 0.5% chlorophyll_a,b_, 0.41 ± 0.05% carotenoids, 12.55 ± 1.6% starch, 3.27 ± 0.7% lipids, 22.24 ± 1.8% ulvan, 13.37 ± 1.5% proteins, and 10.66 ± 0.9% cellulose from the dry weight of *Ulva fasciata* [3]. The obtained results were better than those reported for *Ulva ohnoi* seaweed dry biomass, which yielded 45.42 ± 1.91%, 3.67 ± 1.38%, 3.81 ± 1.26%, 13.88 ± 0.40%, 14.83 ± 1.06%, and 8.70 ± 1.87% of salt-, starch-, lipid-, ulvan-, protein-, and cellulose-rich extracts, respectively, with a total recovery of approximately 90.31 ± 1.94% of *Ulva ohnoi* dry weight [9]. The current reported yields were also comparable to those reported for Indian *Ulva fasciata* seaweed dry biomass, which yielded approximately 26% MRWE, 3% lipids, 25% ulvan, and 11% cellulose [4].

### 3.2. Process Simulation for the Suggested Fully Integrated Process of *Ulva fasciata* Valorization Into Sustainable Value-Added Products

#### 3.2.1. Production of Pigments and Antioxidants

**Figure 2:**
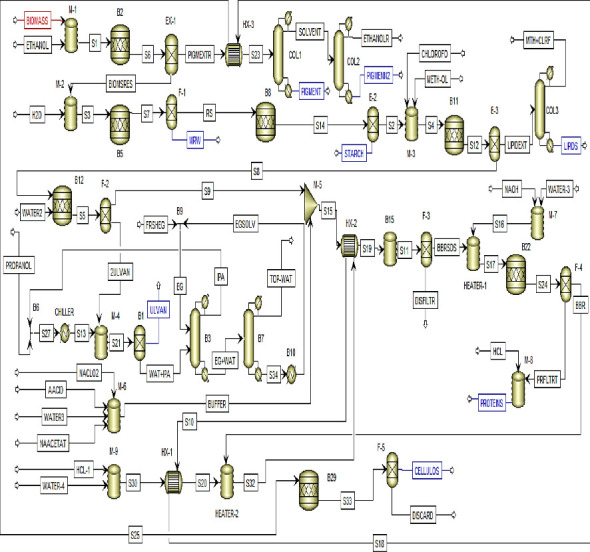
Process simulation diagram for the suggested fully integrated process of *Ulva fasciata* valorization into sustainable value-added products.

Initially, 24 tons per day (t/d) of biomass were combined with absolute ethanol at 60°C for pigment extraction in a ratio of 1:20 (w/v). The pigment extraction stream analysis and properties across designated units are fully illustrated in supporting data Table S5. The resultant pigment extract (PIGMEXTR) stream was directed into COL1, aimed at isolating the primary product, “pigment and antioxidant,” from both the top and bottom products (Figure 2). The top product, identified as “solvent,” underwent recovery through a subsequent distillation column labeled COL2. COL2 separated and discharged ethanol from its top, while pigment and antioxidant residues emerged from its bottom (Figure 2).  Table 3 provides specific details and specifications for COL1 and COL2.

#### 3.2.2. Production of MRWE

Water (H_2_O) was introduced to the BIOMSRES, which exits from the bottom of EX-1, in a ratio of 1:20 (w/v) (BIOMSRES:H_2_O). The MRWE extraction stream analysis and properties across designated units are fully illustrated in supporting data Table S6. The separation processes then extracted MRWE from the bottom of the F-1 unit and directed the top product, known as residue (RS), into subsequent processes (Figure 2).

#### 3.2.3. Production of Starch

For starch extraction, the RS stream was directed toward the hydrolysis reactor (RStoic), as shown in Figure 2. The starch extraction stream analysis and properties across designated units are fully illustrated in supporting data Table S7. The RStoic (B8) was set to operate at a temperature of 40°C, a pressure of 1 atm, and a conversion reaction rate of 20.2% from the initial “RS” input.

#### 3.2.4. Production of Lipids

The BIOMSRES stream (S2) underwent mixing with chloroform and methanol at a ratio of 1:2, as illustrated in Figure 2. The lipid extraction stream analysis and properties across designated units are fully illustrated in supporting data Table S8. The resultant mixed stream (S4) was introduced into the hydrolysis reactor (B11) to facilitate the production of lipid extract. The specifications for reactor B11 involved operating at a temperature of 50°C and a pressure of 1 atm, with reaction conversions specified according to Table 4. To obtain the lipids and recover the chloroform–methanol mixture, a distillation column labeled COL3 was utilized.  Table 3 details the conditions for COL3, which governed the separation process.

#### 3.2.5. Production of Ulvan

The BIOMSRES stream (S8) was mixed with distilled water with a ratio of 1:20 (w/v), as shown in Figure 2. The ulvan extraction stream analysis and properties across designated units are fully illustrated in supporting data Table S9. The filtration process received this mixture (S5) and sent the upper product (S9) to other processes. Then, the filtrate (2ULVAN) was mixed with chilled isopropyl alcohol at −40°C, and the mixing ratio was 1:2.5 (v/v). Following Ulvan's separation, the Radfrac distillation tower (B3) recovered the isopropyl alcohol. However, there is an azeotrope between water and isopropyl alcohol, and the separation of pure isopropanol is very difficult under normal conditions. So, using ethylene glycol as a solvent is an effective solution to the azeotrope problem. The column (B3) recycles the isopropanol as it exits. To recover the ethylene glycol solvent, another distillation column (B7) was used. The recovered ethylene glycol (EGSOLV) was sent back to mixer B9 in order to mix it with fresh ethylene glycol (FRSHEG), and then, the mixture (EG) was fed to the column (B3).  Table 3 displays the specifications for columns B3 and B7.

#### 3.2.6. Production of Proteins

The BIOMSRES stream (S9) underwent a bleaching process utilizing an acetate buffer containing 1% NaClO_2_ at a ratio of 1:40 (w/v) and conducted at a temperature of 60°C. The protein extraction stream analysis and properties across designated units are fully illustrated in supporting data Table S10. The preparation of the buffer solution involved mixing components—acetic acid, sodium acetate, water, and NaClO_2_—using a mixer (M-6). The F-3 unit then filtered the resulting mixture stream (S11), directing the bleached biomass residue (BBR) toward the subsequent neutralization and soaking processes depicted in Figure 2. Following this, the resulting mixture underwent filtration, neutralization, and hydrolysis stages. The hydrolysis, aimed at producing proteins from the biomass, was executed within the B22 unit at a temperature of 60°C and a pressure of 1 atm.

#### 3.2.7. Production of Cellulose

The BBR stream was combined with 5% HCl at a ratio of 1:20 (w/v), as depicted in Figure 2. The cellulose extraction stream analysis and properties across designated units are fully illustrated in supporting data Table S11. The mixture was heated using the heater (HEATER-2) to reach a temperature of 100°C. Subsequently, this heated mixture was directed into the hydrolysis reactor (B29), which was maintained at a temperature of 25°C and a pressure of 1 atm to facilitate the production of cellulose.

### 3.3. Process Heat Integration and Energy Analysis

Three heat exchangers were employed to conserve energy and cut costs. In the process, the S32 product stream, exiting the heater at 100°C with a mass flow rate of 150 tons per day, was utilized to elevate the temperature of stream S15 from 25°C to 60°C in HX-2. Concurrently, stream S10 was employed to increase the temperature of stream S30 from 27°C to 90°C in HX-1. This resulted in a decreased heat duty requirement for HEATER-2. Similarly, stream S18 was utilized in HX-3 to raise the temperature of stream PIGMEXTR from 60°C to 78°C, following the same approach.

The estimation of total energy consumption across various units involved in this simulation is detailed in Table 5. It enumerates the types of heat exchangers and their corresponding units and provides a comprehensive overview of the diverse heat exchange mechanisms and their corresponding types employed throughout the simulation. These units encompass a range of functions, including coolers, heaters, process exchangers, and reboilers. Each unit plays a distinct role in the overall process, contributing to the exchange or regulation of heat within the system.

### 3.4. Economic Evaluation

The investment decisions of manufacturers heavily rely on the overall capital expenditure, particularly the cost associated with the ISBL plant, which stands as a primary consideration. In this context, utilizing Bridgewater's approach, the estimated ISBL cost for the process presented here amounts to $19,488,430.20, as evident in Table 6. Understanding the total production cost, in addition to the ISBL cost, proves critical in determining process feasibility.  Table 7 delineates the costs associated with raw materials and extracted products from *Ulva fasciata*.  Table 8 highlights the overall cost per ton of products at $3107.22. Thus, Tables 6, 7, and 8 collectively emphasize the pivotal role played by both the ISBL cost as an initial metric and the comprehensive production expenditure in evaluating the feasibility of manufacturing processes. This dual perspective provides critical insights into the financial aspects necessary for assessing the viability of such processes.

The NPV represents the disparity between the present values of cash inflows and outflows within a specific timeframe (15 years in this project). When the NPV is positive, a project is considered economically viable.  Table 9 offers an overview of the NPV for this process. Cash flow initiation, known as the design phase, begins in Year 1 of the project. During the construction and installation stages in Year 2, the project allocates the entire fixed capital investment within this phase. Depreciation costs are deducted from the profit starting in Year 3, when the operation reaches full capacity.

The proposed approach, as shown in Table 9, demonstrates promising indicators with an NPV of $49,755,544.90 and an impressive ROI of 485%. In addition, the IRR, calculated at a significant 17%, signals a strong potential for profitability. Moreover, the anticipated payback period of 7 years indicates a swift recovery of the initial capital investment. These findings highlight the financial feasibility and attractiveness of this process initiative. The robust IRR and rapid payback period underscore the project's potential for substantial returns and swift capital recovery, establishing it as an appealing and promising investment opportunity.

## 4. *Ulva fasciata* Value-Added Products and Their Proposed Potential Uses

The present research presents an environmentally friendly, sequential, and zero-waste method to generate various valuable products from *Ulva fasciata*, including 34.89%, 2.61%, 0.41%, 12.55%, 3.27%, 22.24%, 13.37%, and 10.66% of MRWE, chlorophyll_a,b_, carotenoids, starch, lipids, ulvan, proteins, and cellulose, respectively. The produced MRWE can be used as liquid organic fertilizer. The produced chlorophyll and carotenoids have different uses as natural pigments and antioxidants. The production of biodegradable plastics, as well as the biofuels, food, and pharmaceutical industries, can utilize *Ulva*-derived starch and cellulose. Ulvan, with its biocidal activities, can be applied in food packaging, water disinfection, wound healing, and other biomedical applications. The food and animal feed industries can apply *Ulva*-derived lipids and proteins [3, 4, 9].

Thus, the green *Ulva fasciata* biomass can be considered a good candidate for a sustainable blue economy. Moreover, *Ulva* biomass has been reported for carbon dioxide sequestration, and it has the ability to capture about 3.85 million tons of CO_2_ equivalent [25]. For its importance, according to statistics, the worldwide production of cultivated *Ulva* reached 2155 metric tons in 2019, with an estimated value of approximately 750,000 USD [26]. *Ulva* is reported to have wide applications in the existing food, animal feed, and pharmaceutical industrial sectors, in addition to biofuel production and agricultural sector [27, 28]. In addition, incorporating *Ulva* cultivation into integrated multitrophic aquaculture (IMTA) systems has proven its viability for large-scale operations and contributes significantly to environmental sustainability [29–31]. For capitalizing on their potential applications in food production, animal feed, biofuel, and bioremediation, *Ulva* have been cultivated globally using pilot commercial systems [32]. Initial commercial trials took place in America, as a biomass feedstock for biomethane production. In Denmark, research was conducted on outdoor tank cultivation to be used as a feedstock for bioenergy production. In South Africa, *Ulva* are harvested for animal feed production. In addition, in Saudi Arabia, *Ulva* is strategically grown as a biofilter within IMTA systems, while in Australia, land-based cultivation of *Ulva* is undertaken for nutraceuticals and cosmeceuticals industries [32]. In several developing countries, such as the Pacific Islands, Indonesia, Tanzania, and the Philippines, promoting seaweed cultivation has been recommended as a strategy to reduce fishing pressure, alleviate poverty, create new job opportunities, and empower women by providing an alternative income source [33].

## 5. Conclusion

The study concludes that extracting value-added products from *Ulva fasciata* is economically viable and sustainable, as it has several industrial applications. Moreover, key financial indicators—an NPV of $49,755,544.90, an ROI of 485%, and an IRR of 17%—coupled with a 7-year payback period, demonstrate the process's profitability and quick investment recovery.

This proposed study can be utilized for the preliminary design and to build a functional biorefinery for sustainable products derived from marine algal biomass. Further work is planned now to incorporate pilot-scale validations, thereby creating a new market for the bio-based blue economy, which would consider other possible scaling challenges including maintaining product quality, solvent recovery efficiency, and equipment optimization, to align the simulated and real-world results. This approach is both strategic and promising, requiring only three fundamental inputs: carbon dioxide, sunlight, and seawater. It is essential to emphasize that the exact pricing of *Ulva fasciata*–derived bioproducts intended for widespread usage remains undetermined, as they have not yet entered the market and their unique attributes are still unknown. However, this suggested zero-waste BIOMSRES process can improve resource sustainability by converting biomass in the best way possible, lower the growth of invasive algal biomass along the Mediterranean coast, and make the process more useful in low-carbon sectors. It can also lessen the negative impacts of harmful algal blooms on tourism, the environment, and the economy. Further work is being undertaken now to assess the life cycle assessment (LCA), water footprint, and carbon footprint of the suggested bioprocess to assure its economic and environmental viability.

## Figures and Tables

**Table 1:** *Ulva fasciata* biomass composition [3].

Proximate analysis	Weight %
Moisture content	8.5
Volatile content	70.7
Fixed carbon	0.3
Ash content	20.5
Calorific value MJ/kg	15.19

**Cellulose, hemicellulose, and lignin contents**	**Weight %**

Hemicellulose	32.91
Cellulose	9.8
Lignin	1.5

**Chemical constituents**	**Weight %**

Organic C	24.59
Organic matter	42.30
Moisture content	8.5
Total nitrogen	2.1

**Mineral composition**	**mg/kg**

P	33
K	260
Ca	3700
Mg	82.47
Fe	180.14
Zn	66.32
Mn	15.29
Cu	3.23
Ni	0.0519
Cd	0.591
Cr	0.31
Pb	0.0001
As	0.002
Hg	0.001
Co	0.0278

**Table 2:** Overview of economic assumptions and evaluation parameters.

Economic assumptions	Parameters
Equity cost	25%
Debt cost	5%
Capital cost	15%
Debt-to-equity ratio	0.5
Rate of discount	11%
Taxation rate	22.50%
Depreciation method	Falling balance
Depreciation period	5 years
Depreciation rate	10%
Project duration	15 years

**Table 3:** Distillation towers specifications.

Model	COL1	COL2	COL3	B3		B7
Type	DSTWU	DSTWU	DSTWU	RadFrac		DSTWU
Number of stages	7	4	10	30		15
Reflux ratio	0.1240405	0.263377	0.14508	1.2		0.13163
Feed stage	5	3	6	EG	WAT + IPA	8
				4	25	

**Table 4:** Conversion of fatty acid composition in *Ulva fasciata*.

Fatty acid	Content (%)	Total amount in the biomass (kg)	Conversion (%)
Caprylic acid	1.17	0.38259	0.077
Capric acid	0.84	0.27468	0.055
Myristic acid	4.45	1.45515	0.294
Palmitic acid	38.93	12.73011	2.570
Margaric acid	1.63	0.53301	0.108
Stearic acid	3.72	1.21644	0.246
Arachidic acid	0.39	0.12753	0.026
Behenic acid	2.64	0.86328	0.174
Lignoceric acid	0.92	0.30084	0.061
Palmitoleic acid (*n* − 9)	3.08	1.00716	0.203
Heptadecenoic acid	1.84	0.60168	0.121
Oleic acid (*n* − 9)	1.16	0.37932	0.077
Linoleic acid (*n* − 6)	1.42	0.46434	0.094
α-Linolenic acid (*n* − 3)	15.68	5.12736	1.035
*γ*-Linolenic acid (*n* − 6)	2.95	0.96465	0.195
Stearidonic acid (*n* − 3)	16.15	5.28105	1.066
Gondoic acid (*n* − 9)	1.16	0.37932	0.077
Eicosapentaenoic acid (*n* − 3)	1.87	0.61149	0.123
Total	100.00	32.7	6.601

**Table 5:** Energy analysis.

Heat exchanger	Type	Base duty (kW)
B10	Cooler	1129
B11_heat_Exchanger	Heater	391.8
B12_heat_Exchanger	Heater	107.6
B2_heat_Exchanger	Heater	582.5
B22_heat_Exchanger	Heater	474.1
B29_heat_Exchanger	Cooler	222.5
B5_heat_Exchanger	Cooler	311
B8_heat_Exchanger	Heater	493.9
CHILLER	Cooler	46.86
Condenser@B3	Cooler	189.8
Condenser@B7	Cooler	2.08E + 04
Condenser@COL1	Cooler	4211
Condenser@COL2	Cooler	4963
Condenser@COL3	Cooler	40.22
HEATER-1	Heater	38.14
HEATER-2	Heater	4317
Reboiler@B3	Heater	3209
Reboiler@B7	Heater	2.15E + 04
Reboiler@COL1	Heater	1669
Reboiler@COL2	Heater	4963
Reboiler@COL3	Heater	739.2
Total	kW	70448.62

**Table 6:** Summary of the total capital costs.

Cost item	Cost ($)
ISBL	19,488,430.20
OSBL	5,846,529.06
Direct cap. investment (ISBL + OSBL)	25,334,959.26
Contingency cost	2,533,495.93
Engineering cost	3,800,243.89
Fixed capital cost	31,668,699.07
Working capital	3,800,243.89
Startup expenses	3,800,243.89
Total capital investment	49,086,483.56

**Table 7:** Cost breakdown of raw materials and products.

Raw materials				Products			
Parameter	ton/day	$/ton	Total	Parameter	ton/day	$/ton	Total
Biomass	24	1440	34,560	Carotene	0.09547	130,000	12,411.68
Ethanol	0.004134	850	3.5142	Chlorophyll	0.62906	12,000	7548.69
Chloroform	3.87819E − 06	600	0.0023269	MRWE	8.373671	1000	8373.67
Methanol	2.4E − 05	480	0.01152	Cellulose	2.56015	2500	6400.37
Isopropanol	0.1056	1290	136.224	Lipids	0.78483	2100	1648.14
NaClO_2_	2.30688	1200	2768.256	Proteins	3.211071	5000	16,055.35
Acetic acid	0.0816	600	48.96	Starch	3.011624	3000	9034.87
Water	249.215	0.10	24.92151	Ulvan	5.338134	15,000	80,072.01
Sodium acetate	1.781	240	427.49568				
HCl	0.408	5000	2040				
NaOH	0.4608	345	158.976				
Fresh ethylene glycol	0.024	600	14.4				
Total cost $ for (t/d)			40,182.76				141,544.79

**Table 8:** Comprehensive overview of production costs and gross profit.

Cost parameter	Assumption	Value ($)
Raw materials		40,182.76
Energy	$0.16/m^3^	25,427.72
Heat losses	5%	1271.39
Variable production cost/d		66,881.87
Product formulation into beads and packing	1%	668.82
Maintenance	10%	6688.19
Waste steam disposal	0.5%	334.41
Cost/day		74,573.28
Cost/ton		3107.22
Cost/y		24,609,183.52
Income ($/d)		141,544.79
Income ($/y)		46,709,779.25
Profit/d		66,971.50
Profit/y		22,100,595.73
Profit %		89.8

**Table 9:** Financial metrics—net present value (NPV), internal rate of return (IRR), return on investment (ROI), and payback period for the process.

Project life	Investment (%)	Capacity (%)	Dep.	Investment	Ope. expenses	Revenues	Profit	Dep ex	Taxable income	Tax paid	Cash flow	Dis. cash flow	Pre tax	Column 1
1	0	0	0	0	$0.00	$0.00	$0.00	$0.00	$0.00	$0.00	$0.00	$0.00	$0.00	
2	100	0	0	$49,086,483.56	$0.00	$0.00	$0.00	$0.00	$0.00	$0.00	-$49,086,483.56	-$44,222,057.26	$49,086,483.56	
3	0	100	0.2		$28,409,427.41	$46,709,779.25	$18,300,351.84	$3,660,070.37	$14,640,281.47	$3,294,063.33	$15,006,288.51	$12,179,440.39	$11,712,225.18	
4	0	100	0.32		$28,409,427.41	$46,709,779.25	$18,300,351.84	$5,856,112.59	$12,444,239.25	$2,799,953.83	$15,500,398.01	$10,972,468.82	$12,700,444.18	
5	0	100	0.192		$28,409,427.41	$46,709,779.25	$18,300,351.84	$3,513,667.55	$14,786,684.29	$3,327,003.96	$14,973,347.88	$9,885,107.05	$11,646,343.91	
6	0	100	0.1152		$28,409,427.41	$46,709,779.25	$18,300,351.84	$2,108,200.53	$16,192,151.31	$3,643,234.04	$14,657,117.80	$8,905,501.85	$11,013,883.75	
7	0	100	0.1152		$28,409,427.41	$46,709,779.25	$18,300,351.84	$2,108,200.53	$16,192,151.31	$3,643,234.04	$14,657,117.80	$8,022,974.64	$11,013,883.75	
8	0	100	0.0576		$28,409,427.41	$46,709,779.25	$18,300,351.84	$1,054,100.27	$17,246,251.58	$3,880,406.60	$14,419,945.24	$7,227,905.08	$10,539,538.63	
9	0	100	0		$28,409,427.41	$46,709,779.25	$18,300,351.84	$0.00	$18,300,351.84	$4,117,579.16	$14,182,772.68	$6,511,626.20	$10,065,193.51	
10	0	100	0		$28,409,427.41	$46,709,779.25	$18,300,351.84	$0.00	$18,300,351.84	$4,117,579.16	$14,182,772.68	$5,866,329.91	$10,065,193.51	
11	0	100	0		$28,409,427.41	$46,709,779.25	$18,300,351.84	$0.00	$18,300,351.84	$4,117,579.16	$14,182,772.68	$5,284,981.90	$10,065,193.51	
12	0	100	0		$28,409,427.41	$46,709,779.25	$18,300,351.84	$0.00	$18,300,351.84	$4,117,579.16	$14,182,772.68	$4,761,244.95	$10,065,193.51	
13	0	100	0		$28,409,427.41	$46,709,779.25	$18,300,351.84	$0.00	$18,300,351.84	$4,117,579.16	$14,182,772.68	$4,289,409.87	$10,065,193.51	
14	0	100	0		$28,409,427.41	$46,709,779.25	$18,300,351.84	$0.00	$18,300,351.84	$4,117,579.16	$14,182,772.68	$3,864,333.21	$10,065,193.51	
15	0	100	0		$28,409,427.41	$46,709,779.25	$18,300,351.84	$0.00	$18,300,351.84	$4,117,579.16	$14,182,772.68	$3,481,381.27	$10,065,193.51	Discount rate
														11%
							$237,904,5					NPV	$47,030,647.87	$49,755,544.90
					ROI		484.66%							
					IRR			17%			Average annual cash flow		$7,019,438.86	
											Payback		6.992935556	Years

## Data Availability

Data are available from the corresponding author upon request.

## Nomenclature

CCOP: Cash cost of production
COL: Distillation column
DSTWU: Distillation unit
EX-1: Extractor (component separator)
HX: Heat exchanger
IRR: Internal rate of return
ISBLs: Inside battery limits
MRWE: Mineral-rich water extract
NPV: Net present value
NREL: National Renewable Energy Laboratory
NRTL: Nonrandom two-liquid
OSBLs: Outside battery limits
ROI: Return on investment
TEA: Technoeconomic analysis

## Supporting Information

Table S1 illustrates the heater performance metrics and properties across named units.

Table S2 illustrates all data related to process heat exchangers.

Table S3 illustrates the DSTWU performance and the key component recovery across designated units.

Table S4 illustrates the RStoic analysis across the designated units.

Table S5 illustrates the pigment extraction stream analysis and properties across designated units.

Table S6 illustrates the MRWE extraction stream analysis and properties across designated units.

Table S7 illustrates the starch extraction stream analysis and properties across designated units.

Table S8 illustrates the lipid extraction stream analysis and properties across designated units.

Table S9 illustrates the ulvan extraction stream analysis and properties across designated units.

Table S10 illustrates the protein extraction stream analysis and properties across designated units.

Table S11 illustrates the cellulose extraction stream analysis and properties across designated units.
