# Supplementary material for: Eco-Friendly Extraction of Sustainable and Valorized Value-Added Products From Ulva fasciata Macroalgae: A Holistic Technoeconomic Analysis
Source: Int J Biomater. 2025 Feb 26;2025:5811057. doi: 10.1155/ijbm/5811057 (PMC11986915; doi:10.1155/ijbm/5811057)
Supplement: Supporting Information — Additional supporting information can be found online in the Supporting Information section. [file 5811057.f1.docx]

**Eco-Friendly Extraction of Sustainable and Valorized Value-Added Products from *Ulva fasciata* Macroalgae: A Holistic Techno-Economic Analysis**

Nour Sh. El-Gendy^1,2*^, M. Shaaban Sadek^3^, Hussein N. Nassar ^1,2,4^, Ahmad Mustafa^5,2^

^1^Process Design and Development Department, Egyptian Petroleum Research Institute (EPRI), Nasr City, Cairo P.O. Box 11727, Egypt

^2^Center of Excellence, October University for Modern Sciences and Arts (MSA), 6^th^ of October City, Giza P.O. Box 12566, Egypt

^3^Chemical Engineering Department, Faculty of Engineering, Minia University, Minya P.O. Box 61519, Egypt

^4^Biochemistry Program, Faculty of Physical Therapy, October University for Modern Sciences and Arts (MSA), 6^th^ of October City, Giza P.O. Box 12566, Egypt

^5^General Systems Engineering Department, Faculty of Engineering, October University for Modern Sciences and Arts (MSA), Egypt

***Correspondence to:**

Nour Sh. El-Gendy: [nshelgendy@msa.edu.eg](mailto:nshelgendy@msa.edu.eg)

**Table S1: Heater performance metrics and properties across named units**

| Heater | | | | |
| --- | --- | --- | --- | --- |
| Name | **B10** | **CHILLER** | **HEATER-1** | **HEATER-2** |
| Property method | **NRTL** | **NRTL** | **NRTL** | **NRTL** |
| Specified temperature [C] | **50** | **-40** | **60** | **100** |
| Calculated pressure [atm] | 1 | 1 | 1 | 1 |
| Calculated temperature [C] | 50 | -40 | 60 | 100 |
| Calculated vapor fraction | 0 | 0 | 0 | 1 |
| Calculated heat duty [kW] | -1129.47032 | -46.8582093 | 38.139632 | 3530.84021 |
| Net duty [kW] | -1129.47032 | -46.8582093 | 38.139632 | 3530.84021 |

**Table S2: Process heat exchangers: specifications, performance, and heat transfer properties across named units**

| Process heat exchangers | | | |
| --- | --- | --- | --- |
| **Name** | **HX-1** | **HX-2** | **HX-3** |
| Exchanger specification | **90** | **60** | **70** |
| Units of exchanger specification | **C** | **C** | **C** |
| Exchanger area [sqm] |  | **212.3** |  |
| Minimum temperature approach [C] | **1** | **10** | **10** |
| Inlet hot stream temperature [C] | 99.2786306 | 100 | 99.1022128 |
| Inlet hot stream pressure [atm] | 1 | 1 | 1 |
| Inlet hot stream vapor fraction | 0.898935293 | 1 | 0.732550447 |
| Outlet hot stream temperature [C] | 99.1022128 | 99.2786306 | 70 |
| Outlet hot stream pressure [atm] | 1 | 1 | 1 |
| Outlet hot stream vapor fraction | 0.732550447 | 0.898935293 | 0.027045425 |
| Inlet cold stream temperature [C] | 27.3963545 | 24.9999999 | 60 |
| Inlet cold stream pressure [atm] | 1 | 1 | 1 |
| Inlet cold stream vapor fraction | 0.006246001 | 0 | 0 |
| Outlet cold stream temperature [C] | 90 | 60 | 78.3587381 |
| Outlet cold stream pressure [atm] | 1 | 1 | 1 |
| Outlet cold stream vapor fraction | 0.076155229 | 0 | 0.694333198 |
| Heat duty [kW] | 622.747682 | 380.084252 | 2822.38161 |
| Calculated heat duty [kW] | 622.747682 | 380.084252 | 2822.38161 |
| Required exchanger area [sqm] | 23.9984056 | 8.13268855 | 226.659709 |
| Actual exchanger area [sqm] | 23.9984056 | 8.13268855 | 226.659709 |
| Average U (Dirty) [Watt/sqm-K] | 850 | 850 | 850 |
| UA [J/sec-K] | 20398.6448 | 6912.78526 | 192660.752 |
| LMTD (Corrected) [C] | 30.5288752 | 54.9827945 | 14.6494892 |
| LMTD correction factor | 1 | 1 | 1 |
| Number of shells in series | 1 | 1 | 1 |

**Table S3: Distillation unit (DSTWU) performance and key component recovery across designated units**

| DSTWU | | | | |
| --- | --- | --- | --- | --- |
| **Name** | **B7** | **COL1** | **COL2** | **COL3** |
| Light key component recovery | 0.99999 | 0.99999 | 0.999999 | 0.99999 |
| Heavy key component recovery | 0.00001 | 0.00001 | 0.0000001 | 0.000001 |
| Minimum reflux ratio | 0.033811 | 0.000499 | 0.00000002 | 0.006214 |
| Actual reflux ratio | 0.131626 | 0.124040 | 0.263377 | 0.145080 |
| Minimum number of stages | 6.618502 | 3.105309 | 1.898085 | 4.780263 |
| Number of actual stage | 15 | 7 | 4 | 10 |
| Feed stage | 7.066 | 4.998 | 2.837 | 5.178 |
| Number of actual stage above feed | 6.066 | 3.998 | 1.837 | 4.178 |
| Distillate temperature [C] | 100.016267 | 78.350538 | 78.349990 | 60.31553 |
| Bottom temperature [C] | 197.201646 | 149.610598 | 188.465896 | 220.76686 |
| Distillate to feed fraction | 0.926114 | 0.999906 | 0.999977 | 0.966281 |

**Table S4: RStoic analysis across designated units**

| RStoic | | | | | | | |
| --- | --- | --- | --- | --- | --- | --- | --- |
| **Name** | **B2** | **B5** | **B8** | **B11** | **B12** | **B22** | **B29** |
| Specified pressure [atm] | 1 | 1 | 1 | 1 | 1 | 1 | 1 |
| Specified temperature [C] | 60 | 25 | 40 | 50 | 25 | 60 | 25 |
| Outlet temperature [C] | 60 | 25 | 40 | 50 | 25 | 60 | 25 |
| Outlet pressure [atm] | 1 | 1 | 1 | 1 | 1 | 1 | 1 |
| Calculated heat duty [kW] | 582.4704 | -310.9791 | 493.9214 | 391.8315 | 107.6391 | 474.0959 | -222.4558 |
| Net heat duty [kW] | 582.4704 | -310.9791 | 493.9214 | 391.8315 | 107.6391 | 474.0959 | -222.4558 |
| Calculated vapor fraction | 0.0000 | 0.0000 | 0.0000 | 0.0000 | 0.0000 | 0.0000 | 0.0041 |

**Table S5: Pigment extraction stream analysis and properties across designated units**

| **Stream Name** | **Units** | **BIOMASS** | **ETHANOL** | | **S1** | **S6** | **PIGMEXTR** | **S12** | **BIOMSRES** | **PIGMEXTR3** | **S23** | **PIGMENT** | **SOLVENT** | **PIGMENN2** | **ETHANOLR** |
| --- | --- | --- | --- | --- | --- | --- | --- | --- | --- | --- | --- | --- | --- | --- | --- |
| From |  |  |  | M-1 | | B2 | EX-1 | M-1 | EX-1 | EX-1 | HX-3 | COL1 | COL1 | COL2 | COL2 |
| To |  | M-1 | M-1 | B2 | | EX-1 | HX-3 | B2 | M-2 | HX-3 | COL1 |  | COL2 |  |  |
| Stream Class |  | MIXCINC | MIXCINC | MIXCINC | | MIXCINC | MIXCINC | MIXCINC | MIXCINC | MIXCINC | MIXCINC | MIXCINC | MIXCINC | MIXCINC | MIXCINC |
| Temperature | C | 25 | 25 | 25 | | 60 | 60 | 25 | 60 | 60 | 78.3587 | 149.611 | 78.3505 | 188.466 | 78.35 |
| Pressure | atm | 1 | 1 | 1 | | 1 | 1 | 1 | 1 | 1 | 1 | 1 | 1 | 1 | 1 |
| Mass Vapor Fraction |  | 0 | 0 | 0 | | 0 | 0 | 0 | 0 | 0 | 0.693082 | 0 | 0 | 0 | 0 |
| Mass Liquid Fraction |  | 0 | 1 | 0.940001 | | 0.941812 | 1 | 0.940001 | 0 | 1 | 0.306918 | 1 | 1 | 1 | 1 |
| Mass Solid Fraction |  | 1 | 0 | 0.0599991 | | 0.0581878 | 0 | 0.0599991 | 1 | 0 | 0 | 0 | 0 | 0 | 0 |
| Mass Enthalpy | J/kg | -1.15E+07 | -6.04E+06 | -6.37E+06 | | -6.24E+06 | -5.92E+06 | -6.37E+06 | -1.15E+07 | -5.92E+06 | -5.27E+06 | -971260 | -5.86E+06 | 392507 | -5.86E+06 |
| Mass Density | kg/cum | 2153.63 | 783.345 | 814.437 | | 767.726 | 738.37 | 814.437 | 2153.63 | 738.37 | 2.30221 | 97.4231 | 721.286 | 501.14 | 721.416 |
| Enthalpy Flow | kW | -3196.97 | -26279.4 | -29476.3 | | -28893.5 | -25793.4 | -29476.3 | -3100.17 | -25793.4 | -22971 | -7.07151 | -25506.4 | 0.433731 | -25506.6 |
| Mass Flows | kg/hr | 1000 | 15666.9 | 16666.9 | | 16666.7 | 15696.9 | 16666.9 | 969.8 | 15696.9 | 15696.9 | 26.2107 | 15670.7 | 3.9781 | 15666.7 |
| BIOMASS | kg/hr | 1000 | 0 | 1000 | | 969.8 | 0 | 1000 | 969.8 | 0 | 0 | 0 | 0 | 0 | 0 |
| ETHANOL | kg/hr | 0 | 15666.9 | 15666.9 | | 15666.9 | 15666.9 | 15666.9 | 0 | 15666.9 | 15666.9 | 0.156669 | 15666.8 | 0.0156668 | 15666.7 |
| CLROFYLB | kg/hr | 0 | 0 | 0 | | 26.0543 | 26.0543 | 0 | 0 | 26.0543 | 26.0543 | 26.0541 | 0.000260543 | 0.000189191 | 7.14E-05 |
| CAROTENE | kg/hr | 0 | 0 | 0 | | 3.96225 | 3.96225 | 0 | 0 | 3.96225 | 3.96225 | 3.80E-14 | 3.96225 | 3.96225 | 3.96E-07 |
| Mass Fractions |  |  |  |  | |  |  |  |  |  |  |  |  |  |  |
| BIOMASS |  | 1 | 0 | 0.0599991 | | 0.0581878 | 0 | 0.0599991 | 1 | 0 | 0 | 0 | 0 | 0 | 0 |
| ETHANOL |  | 0 | 1 | 0.940001 | | 0.940011 | 0.998088 | 0.940001 | 0 | 0.998088 | 0.998088 | 0.00597729 | 0.999747 | 0.00393825 | 1 |
| CLROFYLB |  | 0 | 0 | 0 | | 0.00156325 | 0.00165984 | 0 | 0 | 0.00165984 | 0.00165984 | 0.994023 | 1.66E-08 | 4.76E-05 | 4.55E-09 |
| CAROTENE |  | 0 | 0 | 0 | | 0.000237734 | 0.000252422 | 0 | 0 | 0.000252422 | 0.000252422 | 1.45E-15 | 0.000252844 | 0.996014 | 2.53E-11 |

**Table S6: MRWE extraction stream analysis and properties across designated units**

| **Stream Name** | **Units** | **BIOMSRES** | **H2O** | **S3** | **S7** | **MRWE** |
| --- | --- | --- | --- | --- | --- | --- |
| From |  | EX-1 |  | M-2 | B5 | F-1 |
| To |  | M-2 | M-2 | B5 | F-1 |  |
| Stream Class | | MIXCINC | MIXCINC | MIXCINC | MIXCINC | MIXCINC |
| Temperature | C | 60 | 20 | 20.013 | 25 | 25 |
| Pressure | atm | 1 | 1 | 1 | 1 | 1 |
| Mass Vapor Fraction | | 0 | 0 | 0 | 0 | 2.61E-05 |
| Mass Liquid Fraction | | 0 | 1 | 0.953657 | 0.970329 | 0.999974 |
| Mass Solid Fraction | | 1 | 0 | 0.0463433 | 0.0296706 | 0 |
| Mass Enthalpy | J/kg | -1.15E+07 | -1.59E+07 | -1.57E+07 | -1.57E+07 | -1.59E+07 |
| Mass Density | kg/cum | 2153.63 | 997.832 | 1023.27 | 1009.16 | 981.153 |
| Enthalpy Flow | kW | -3100.17 | -88072.9 | -91173.1 | -91484.1 | -1537.68 |
| Mass Flows | kg/hr | 969.8 | 19956.6 | 20926.4 | 20926.4 | 348.903 |
| BIOMASS | kg/hr | 969.8 | 0 | 969.8 | 620.9 | 0 |
| WATER | kg/hr | 0 | 19956.6 | 19956.6 | 20305.5 | 348.864 |
| P | kg/hr | 0 | 0 | 0 | 0.00142246 | 0.00142246 |
| K | kg/hr | 0 | 0 | 0 | 0.00841844 | 0.00841844 |
| CA | kg/hr | 0 | 0 | 0 | 0.000351094 | 0.000351094 |
| MG | kg/hr | 0 | 0 | 0 | 0.000118107 | 0.000118107 |
| FE | kg/hr | 0 | 0 | 0 | 0.00506957 | 0.00506957 |
| ZN | kg/hr | 0 | 0 | 0 | 0.0118776 | 0.0118776 |
| MN | kg/hr | 0 | 0 | 0 | 0.010427 | 0.010427 |
| CU | kg/hr | 0 | 0 | 0 | 0.00016027 | 0.00016027 |
| NI | kg/hr | 0 | 0 | 0 | 1.23E-06 | 1.23E-06 |
| CD | kg/hr | 0 | 0 | 0 | 0.000122367 | 0.000122367 |
| CR | kg/hr | 0 | 0 | 0 | 1.09E-06 | 1.09E-06 |
| PB | kg/hr | 0 | 0 | 0 | 7.23E-07 | 7.23E-07 |
| AS | kg/hr | 0 | 0 | 0 | 2.61E-08 | 2.61E-08 |
| HG | kg/hr | 0 | 0 | 0 | 7.00E-08 | 7.00E-08 |
| COBALT | kg/hr | 0 | 0 | 0 | 0.000680594 | 0.000680594 |
| Mass Fractions | |  |  |  |  |  |
| BIOMASS |  | 1 | 0 | 0.0463433 | 0.0296706 | 0 |
| WATER |  | 0 | 1 | 0.953657 | 0.970328 | 0.999889 |
| P |  | 0 | 0 | 0 | 6.80E-08 | 4.08E-06 |
| K |  | 0 | 0 | 0 | 4.02E-07 | 2.41E-05 |
| CA |  | 0 | 0 | 0 | 1.68E-08 | 1.01E-06 |
| MG |  | 0 | 0 | 0 | 5.64E-09 | 3.39E-07 |
| FE |  | 0 | 0 | 0 | 2.42E-07 | 1.45E-05 |
| ZN |  | 0 | 0 | 0 | 5.68E-07 | 3.40E-05 |
| MN |  | 0 | 0 | 0 | 4.98E-07 | 2.99E-05 |
| CU |  | 0 | 0 | 0 | 7.66E-09 | 4.59E-07 |
| NI |  | 0 | 0 | 0 | 5.87E-11 | 3.52E-09 |
| CD |  | 0 | 0 | 0 | 5.85E-09 | 3.51E-07 |
| CR |  | 0 | 0 | 0 | 5.20E-11 | 3.12E-09 |
| PB |  | 0 | 0 | 0 | 3.45E-11 | 2.07E-09 |
| AS |  | 0 | 0 | 0 | 1.25E-12 | 7.49E-11 |
| HG |  | 0 | 0 | 0 | 3.34E-12 | 2.01E-10 |
| COBALT |  | 0 | 0 | 0 | 3.25E-08 | 1.95E-06 |

**Table S7: Starch extraction stream analysis and properties across designated units**

| **Stream Name** | **Units** | **RS** | **S14** | **STARCH** |
| --- | --- | --- | --- | --- |
| From |  | F-1 | B8 | E-2 |
| To |  | B8 | E-2 |  |
| Stream Class |  | MIXCINC | MIXCINC | MIXCINC |
| Temperature | C | 25 | 40 | 40 |
| Pressure | atm | 1 | 1 | 1 |
| Mass Vapor Fraction |  | 0 | 0 | 0 |
| Mass Liquid Fraction |  | 0.969826 | 0.969827 | 0 |
| Mass Solid Fraction |  | 0.0301737 | 0.0301729 | 1 |
| Mass Enthalpy | J/kg | -1.57E+07 | -1.56E+07 | -7.05E+06 |
| Mass Density | kg/cum | 1009.44 | 993.635 | 1505.4 |
| Enthalpy Flow | kW | -89946.4 | -89452.4 | -245.705 |
| Mass Flows | kg/hr | 20577.5 | 20577.5 | 125.484 |
| BIOMASS | kg/hr | 620.9 | 495.4 | 0 |
| WATER | kg/hr | 19956.6 | 19956.6 | 0 |
| STARCH | kg/hr | 0 | 125.484 | 125.484 |
| Mass Fractions |  |  |  |  |
| BIOMASS |  | 0.0301737 | 0.0240748 | 0 |
| WATER |  | 0.969826 | 0.969827 | 0 |
| STARCH |  | 0 | 0.00609812 | 1 |

**Table S8: Lipids extraction stream analysis and properties across designated units**

| **Stream Name** | **Units** | **S2** | **CHLOROFO** | **METH-OL** | **S4** | **S12** | **LIPIDEXT** | **MTH+CLRF** | **LIPIDS** |
| --- | --- | --- | --- | --- | --- | --- | --- | --- | --- |
| From |  | E-2 |  |  | M-3 | B11 | E-3 | COL3 | COL3 |
| To |  | M-3 | M-3 | M-3 | B11 | E-3 | COL3 |  |  |
| Stream Class |  | MIXCINC | MIXCINC | MIXCINC | MIXCINC | MIXCINC | MIXCINC | MIXCINC | MIXCINC |
| Temperature | C | 40 | 25 | 25 | 40.0794 | 50 | 50 | 60.3155 | 220.768 |
| Pressure | atm | 1 | 1 | 1 | 1 | 1 | 1 | 1 | 1 |
| Mass Vapor Fraction |  | 0 | 0 | 0 | 0 | 0 | 0 | 0 | 0 |
| Mass Liquid Fraction |  | 0.975777 | 1 | 1 | 0.975954 | 0.977541 | 1 | 1 | 1 |
| Mass Solid Fraction |  | 0.0242225 | 0 | 0 | 0.0240462 | 0.0224589 | 0 | 0 | 0 |
| Mass Enthalpy | J/kg | -1.57E+07 | -1.11E+06 | -7.47E+06 | -1.56E+07 | -1.56E+07 | -3.18E+06 | -5.25E+06 | 8.33E+07 |
| Mass Density | kg/cum | 991.567 | 1479.62 | 784.968 | 990.953 | 979.592 | 883.484 | 893.756 | 760.481 |
| Enthalpy Flow | kW | -89206.7 | -15.4488 | -207.508 | -89429.7 | -89037.8 | -161.335 | -218.881 | 756.51 |
| Mass Flows | kg/hr | 20452 | 50 | 100 | 20602 | 20602 | 182.7 | 149.999 | 32.7012 |
| BIOMASS | kg/hr | 495.4 | 0 | 0 | 495.4 | 462.7 | 0 | 0 | 0 |
| WATER | kg/hr | 19956.6 | 0 | 0 | 19956.6 | 19956.6 | 0 | 0 | 0 |
| C8:0 | kg/hr | 0 | 0 | 0 | 0 | 0.38259 | 0.38259 | 0 | 0.38259 |
| C10:0 | kg/hr | 0 | 0 | 0 | 0 | 0.27468 | 0.27468 | 0 | 0.27468 |
| C14:0 | kg/hr | 0 | 0 | 0 | 0 | 1.45515 | 1.45515 | 0 | 1.45515 |
| C16:0 | kg/hr | 0 | 0 | 0 | 0 | 12.7301 | 12.7301 | 0 | 12.7301 |
| C17:0 | kg/hr | 0 | 0 | 0 | 0 | 0.53301 | 0.53301 | 0 | 0.53301 |
| C18:0 | kg/hr | 0 | 0 | 0 | 0 | 1.21644 | 1.21644 | 0 | 1.21644 |
| C20:0 | kg/hr | 0 | 0 | 0 | 0 | 0.12753 | 0.12753 | 0 | 0.12753 |
| C22:0 | kg/hr | 0 | 0 | 0 | 0 | 0.86328 | 0.86328 | 0 | 0.86328 |
| C24:0 | kg/hr | 0 | 0 | 0 | 0 | 0.30084 | 0.30084 | 0 | 0.30084 |
| C16:1 | kg/hr | 0 | 0 | 0 | 0 | 1.00716 | 1.00716 | 0 | 1.00716 |
| C17:1 | kg/hr | 0 | 0 | 0 | 0 | 0.60168 | 0.60168 | 0 | 0.60168 |
| C18:1 | kg/hr | 0 | 0 | 0 | 0 | 0.37932 | 0.37932 | 0 | 0.37932 |
| C18:2 | kg/hr | 0 | 0 | 0 | 0 | 0.46434 | 0.46434 | 0 | 0.46434 |
| C18:3- | kg/hr | 0 | 0 | 0 | 0 | 5.12736 | 5.12736 | 0 | 5.12736 |
| C18:3 | kg/hr | 0 | 0 | 0 | 0 | 0.96465 | 0.96465 | 0 | 0.96465 |
| C18:4 | kg/hr | 0 | 0 | 0 | 0 | 5.28105 | 5.28105 | 0 | 5.28105 |
| C20:1 | kg/hr | 0 | 0 | 0 | 0 | 0.37932 | 0.37932 | 0 | 0.37932 |
| C20:5 | kg/hr | 0 | 0 | 0 | 0 | 0.61149 | 0.61149 | 6.11E-07 | 0.611489 |
| METHANOL | kg/hr | 0 | 0 | 100 | 100 | 100 | 100 | 99.999 | 0.001 |
| CLORFORM | kg/hr | 0 | 50 | 0 | 50 | 50 | 50 | 49.9998 | 0.000161591 |
| Mass Fractions |  |  |  |  |  |  |  |  |  |
| BIOMASS |  | 0.0242225 | 0 | 0 | 0.0240462 | 0.0224589 | 0 | 0 | 0 |
| ETHANOL |  | 0 | 0 | 0 | 0 | 0 | 0 | 0 | 0 |
| WATER |  | 0.975777 | 0 | 0 | 0.968673 | 0.968673 | 0 | 0 | 0 |
| C8:0 |  | 0 | 0 | 0 | 0 | 1.86E-05 | 0.00209409 | 0 | 0.0116996 |
| C10:0 |  | 0 | 0 | 0 | 0 | 1.33E-05 | 0.00150345 | 0 | 0.0083997 |
| C14:0 |  | 0 | 0 | 0 | 0 | 7.06E-05 | 0.0079647 | 0 | 0.0444984 |
| C16:0 |  | 0 | 0 | 0 | 0 | 0.000617905 | 0.0696777 | 0 | 0.389286 |
| C17:0 |  | 0 | 0 | 0 | 0 | 2.59E-05 | 0.00291741 | 0 | 0.0162994 |
| C18:0 |  | 0 | 0 | 0 | 0 | 5.90E-05 | 0.00665813 | 0 | 0.0371987 |
| C20:0 |  | 0 | 0 | 0 | 0 | 6.19E-06 | 0.00069803 | 0 | 0.00389986 |
| C22:0 |  | 0 | 0 | 0 | 0 | 4.19E-05 | 0.00472512 | 0 | 0.0263991 |
| C24:0 |  | 0 | 0 | 0 | 0 | 1.46E-05 | 0.00164663 | 0 | 0.00919967 |
| C16:1 |  | 0 | 0 | 0 | 0 | 4.89E-05 | 0.00551264 | 0 | 0.0307989 |
| C17:1 |  | 0 | 0 | 0 | 0 | 2.92E-05 | 0.00329327 | 0 | 0.0183993 |
| C18:1 |  | 0 | 0 | 0 | 0 | 1.84E-05 | 0.00207619 | 0 | 0.0115996 |
| C18:2 |  | 0 | 0 | 0 | 0 | 2.25E-05 | 0.00254154 | 0 | 0.0141995 |
| C18:3- |  | 0 | 0 | 0 | 0 | 0.000248876 | 0.0280644 | 0 | 0.156794 |
| C18:3 |  | 0 | 0 | 0 | 0 | 4.68E-05 | 0.00527997 | 0 | 0.029499 |
| C18:4 |  | 0 | 0 | 0 | 0 | 0.000256336 | 0.0289056 | 0 | 0.161494 |
| C20:1 |  | 0 | 0 | 0 | 0 | 1.84E-05 | 0.00207619 | 0 | 0.0115996 |
| C20:5 |  | 0 | 0 | 0 | 0 | 2.97E-05 | 0.00334696 | 4.08E-09 | 0.0186993 |
| METHANOL |  | 0 | 0 | 1 | 0.00485389 | 0.00485389 | 0.547345 | 0.666665 | 3.06E-05 |
| CLORFORM |  | 0 | 1 | 0 | 0.00242694 | 0.00242694 | 0.273673 | 0.333335 | 4.94E-06 |

**Table S9: Ulvan extraction stream analysis and properties across designated units**

| **Stream Name** | **Units** | **S8** | **WATER2** | **S5** | **2ULVAN** | **PROPANOL** | **S27** | **S13** | **S21** | **ULVAN** | **WAT+IPA** | **FRSHEG** | **EGSOLV** | **EG** | **IPA** | **EG+WAT** | **TOP-WAT** | **S34** |
| --- | --- | --- | --- | --- | --- | --- | --- | --- | --- | --- | --- | --- | --- | --- | --- | --- | --- | --- |
| From |  | E-3 |  | B12 | F-2 |  | B6 | CHILLER | M-4 | B1 | B1 |  | B10 | B9 | B3 | B3 | B7 | B7 |
| To |  | B12 | B12 | F-2 | M-4 | B6 | CHILLER | M-4 | B1 |  | B3 | B9 | B9 | B3 | B6 | B7 |  | B10 |
| Stream Class |  | MIXCINC | MIXCINC | MIXCINC | MIXCINC | MIXCINC | MIXCINC | MIXCINC | MIXCINC | MIXCINC | MIXCINC | MIXCINC | MIXCINC | MIXCINC | MIXCINC | MIXCINC | MIXCINC | MIXCINC |
| Temperature | C | 50 | 25 | 25 | 24.9884 | 25 | 81.7563 | -40 | 24.8696 | 24.8696 | 24.8696 | 25 | 50 | 49.9973 | 82.2458 | 102.176 | 100.016 | 197.202 |
| Pressure | atm | 1 | 1 | 1 | 1 | 1 | 1 | 1 | 1 | 1 | 1 | 1 | 1 | 1 | 1 | 1 | 1 | 1 |
| Mass Vapor Fraction |  | 0 | 0 | 0 | 0.00104474 | 0 | 0 | 0 | 0.000994901 | 0.378871 | 0 | 0 | 0 | 0 | 0 | 1.10E-07 | 0 | 0 |
| Mass Liquid Fraction |  | 0.97734 | 1 | 0.991885 | 0.998955 | 1 | 1 | 1 | 0.999005 | 0.621129 | 1 | 1 | 1 | 1 | 1 | 1 | 1 | 1 |
| Mass Solid Fraction |  | 0.0226599 | 0 | 0.00811507 | 0 | 0 | 0 | 0 | 0 | 0 | 0 | 0 | 0 | 0 | 0 | 5.59E-09 | 0 | 0 |
| Mass Enthalpy | J/kg | -1.57E+07 | -1.59E+07 | -1.57E+07 | -1.58E+07 | -5.29E+06 | -5.11E+06 | -5.47E+06 | -1.56E+07 | -581908 | -1.57E+07 | -7.40E+06 | -7.32E+06 | -7.32E+06 | -5.11E+06 | -1.37E+07 | -1.55E+07 | -6.82E+06 |
| Mass Density | kg/cum | 980.841 | 993.029 | 912.91 | 520.981 | 780.159 | 711.663 | 849.858 | 531.732 | 3.36897 | 989.45 | 1108.56 | 1081.95 | 1081.95 | 711.029 | 936.225 | 917.418 | 903.503 |
| Enthalpy Flow | kW | -88873.2 | -40503.8 | -129269 | -128501 | -6.41213 | -670.511 | -717.369 | -129219 | -35.953 | -129183 | -2.0551 | -16299.8 | -16300 | -664.098 | -141800 | -125902 | -15170.4 |
| Mass Flows | kg/hr | 20419.3 | 9189.49 | 29608.8 | 29368.6 | 4.36391 | 472.459 | 472.459 | 29841 | 222.425 | 29618.6 | 1 | 8011.08 | 8011.16 | 468.095 | 37161.7 | 29150.6 | 8011.08 |
| BIOMASS | kg/hr | 462.7 | 0 | 240.278 | 0 | 0 | 0 | 0 | 0 | 0 | 0 | 0 | 0 | 0 | 0 | 0 | 0 | 0 |
| WATER | kg/hr | 19956.6 | 9189.49 | 29146.1 | 29146.1 | 0 | 0.045594 | 0.045594 | 29146.2 | 0 | 29146.2 | 0 | 0.291464 | 0.291235 | 0.045594 | 29146.4 | 29146.1 | 0.291464 |
| K | kg/hr | 0 | 0 | 18.815 | 18.815 | 0 | 0 | 0 | 18.815 | 18.815 | 0 | 0 | 0 | 0 | 0 | 0 | 0 | 0 |
| CA | kg/hr | 0 | 0 | 7.05008 | 7.05008 | 0 | 0 | 0 | 7.05008 | 7.05008 | 0 | 0 | 0 | 0 | 0 | 0 | 0 | 0 |
| MG | kg/hr | 0 | 0 | 7.09456 | 7.09456 | 0 | 0 | 0 | 7.09456 | 7.09456 | 0 | 0 | 0 | 0 | 0 | 0 | 0 | 0 |
| FE | kg/hr | 0 | 0 | 1.112 | 1.112 | 0 | 0 | 0 | 1.112 | 1.112 | 0 | 0 | 0 | 0 | 0 | 0 | 0 | 0 |
| C | kg/hr | 0 | 0 | 37.0074 | 37.0074 | 0 | 0 | 0 | 37.0074 | 37.0074 | 0 | 0 | 0 | 0 | 0 | 0 | 0 | 0 |
| O2 | kg/hr | 0 | 0 | 76.6613 | 76.6613 | 0 | 0 | 0 | 76.6613 | 76.6613 | 0 | 0 | 0 | 0 | 0 | 0 | 0 | 0 |
| S | kg/hr | 0 | 0 | 49.3283 | 49.3283 | 0 | 0 | 0 | 49.3283 | 49.3283 | 0 | 0 | 0 | 0 | 0 | 0 | 0 | 0 |
| N | kg/hr | 0 | 0 | 6.7832 | 6.7832 | 0 | 0 | 0 | 6.7832 | 6.7832 | 0 | 0 | 0 | 0 | 0 | 0 | 0 | 0 |
| NA | kg/hr | 0 | 0 | 18.5704 | 18.5704 | 0 | 0 | 0 | 18.5704 | 18.5704 | 0 | 0 | 0 | 0 | 0 | 0 | 0 | 0 |
| ISOPR-01 | kg/hr | 0 | 0 | 0 | 0 | 4.36391 | 472.411 | 472.411 | 472.411 | 0 | 472.411 | 0 | 2.43E-11 | 4.85E-10 | 468.047 | 4.36385 | 4.36385 | 2.43E-11 |
| EG | kg/hr | 0 | 0 | 0 | 0 | 0 | 0.00248838 | 0.00248838 | 0.00248838 | 0.00248838 | 0 | 1 | 8010.79 | 8010.87 | 0.00248838 | 8010.87 | 0.0801087 | 8010.79 |
| Mass Fractions |  |  |  |  |  |  |  |  |  |  |  |  |  |  |  |  |  |  |
| BIOMASS |  | 0.0226599 | 0 | 0.00811507 | 0 | 0 | 0 | 0 | 0 | 0 | 0 | 0 | 0 | 0 | 0 | 0 | 0 | 0 |
| ETHANOL |  | 0 | 0 | 0 | 0 | 0 | 0 | 0 | 0 | 0 | 0 | 0 | 0 | 0 | 0 | 0 | 0 | 0 |
| WATER |  | 0.97734 | 1 | 0.984373 | 0.992427 | 0 | 9.65E-05 | 9.65E-05 | 0.976715 | 0 | 0.98405 | 0 | 3.64E-05 | 3.64E-05 | 9.74E-05 | 0.784314 | 0.999848 | 3.64E-05 |
| K |  | 0 | 0 | 0.000635454 | 0.000640653 | 0 | 0 | 0 | 0.000630509 | 0.0845906 | 0 | 0 | 0 | 0 | 0 | 0 | 0 | 0 |
| CA |  | 0 | 0 | 0.000238107 | 0.000240055 | 0 | 0 | 0 | 0.000236255 | 0.0316965 | 0 | 0 | 0 | 0 | 0 | 0 | 0 | 0 |
| MG |  | 0 | 0 | 0.00023961 | 0.00024157 | 0 | 0 | 0 | 0.000237745 | 0.0318965 | 0 | 0 | 0 | 0 | 0 | 0 | 0 | 0 |
| FE |  | 0 | 0 | 3.76E-05 | 3.79E-05 | 0 | 0 | 0 | 3.73E-05 | 0.00499944 | 0 | 0 | 0 | 0 | 0 | 0 | 0 | 0 |
| C |  | 0 | 0 | 0.00124988 | 0.0012601 | 0 | 0 | 0 | 0.00124015 | 0.166382 | 0 | 0 | 0 | 0 | 0 | 0 | 0 | 0 |
| O2 |  | 0 | 0 | 0.00258914 | 0.00261032 | 0 | 0 | 0 | 0.00256899 | 0.344662 | 0 | 0 | 0 | 0 | 0 | 0 | 0 | 0 |
| S |  | 0 | 0 | 0.001666 | 0.00167963 | 0 | 0 | 0 | 0.00165304 | 0.221775 | 0 | 0 | 0 | 0 | 0 | 0 | 0 | 0 |
| N |  | 0 | 0 | 0.000229094 | 0.000230968 | 0 | 0 | 0 | 0.000227311 | 0.0304966 | 0 | 0 | 0 | 0 | 0 | 0 | 0 | 0 |
| NA |  | 0 | 0 | 0.000627191 | 0.000632323 | 0 | 0 | 0 | 0.000622311 | 0.0834907 | 0 | 0 | 0 | 0 | 0 | 0 | 0 | 0 |
| ISOPR-01 |  | 0 | 0 | 0 | 0 | 1 | 0.999898 | 0.999898 | 0.0158309 | 0 | 0.0159498 | 0 | 3.03E-15 | 6.06E-14 | 0.999897 | 0.000117429 | 0.0001497 | 3.03E-15 |
| EG |  | 0 | 0 | 0 | 0 | 0 | 5.27E-06 | 5.27E-06 | 8.34E-08 | 1.12E-05 | 0 | 1 | 0.999964 | 0.999964 | 5.32E-06 | 0.215568 | 2.75E-06 | 0.999964 |

**Table S10: Proteins extraction stream analysis and properties across designated units**

| **Stream Name** | **Units** | **NACLO2** | **WATER3** | **NAACETAT** | **AACID** | **BUFFER** | **S9** | **S15** | **S19** | **DISFILTR** | **BBRSDS** | **NAOH** | **WATER32** | **S17** | **S24** | **PRFLTRT** | **HCL** | **PROTEINS** |
| --- | --- | --- | --- | --- | --- | --- | --- | --- | --- | --- | --- | --- | --- | --- | --- | --- | --- | --- |
| From |  |  |  |  |  | M-6 | F-2 | M-5 | HX-2 | F-3 | F-3 |  |  | HEATER-1 | B22 | F-4 |  | M-8 |
| To |  | M-6 | M-6 | M-6 | M-6 | M-5 | M-5 | HX-2 | B15 |  | HEATER-1 | M-7 | M-6 | B22 | F-4 | M-8 | M-8 |  |
| Stream Class |  | MIXCINC | MIXCINC | MIXCINC | MIXCINC | MIXCINC | MIXCINC | MIXCINC | MIXCINC | MIXCINC | MIXCINC | MIXCINC | MIXCINC | MIXCINC | MIXCINC | MIXCINC | MIXCINC | MIXCINC |
| Temperature | C | 25 | 25 | 25 | 25 | 25 | 24.9884 | 25 | 60 | 60 | 60 | 25 | 25 | 60 | 60 | 60 | 25 | 59.6501 |
| Pressure | atm | 1 | 1 | 1 | 1 | 1 | 1 | 1 | 1 | 1 | 1 | 1 | 1 | 1 | 1 | 1 | 1 | 1 |
| Mass Vapor Fraction |  | 0 | 0 | 0 | 0 | 0 | 0 | 0 | 0 | 0 | 0 | 0 | 0 | 0 | 0 | 0 | 1 | 0.00356705 |
| Mass Liquid Fraction |  | 1 | 1 | 1 | 1 | 1 | 0 | 0.975612 | 0.975612 | 1 | 0 | 1 | 1 | 0.800629 | 0.911564 | 1 | 0 | 0.996433 |
| Mass Solid Fraction |  | 0 | 0 | 0 | 0 | 0 | 1 | 0.024388 | 0.024388 | 0 | 1 | 0 | 0 | 0.199371 | 0.0884356 | 0 | 0 | 0 |
| Mass Enthalpy | J/kg | -348998 | -1.59E+07 | -7.95E+06 | -7.70E+06 | -1.56E+07 | -1.15E+07 | -1.55E+07 | -1.54E+07 | -1.55E+07 | -1.15E+07 | -10504.8 | -1.59E+07 | -1.46E+07 | -1.32E+07 | -1.29E+07 | -2.53E+06 | -1.27E+07 |
| Mass Density | kg/cum | 686.581 | 993.029 | 898.132 | 1042.7 | 988.567 | 2153.63 | 1001.78 | 967.049 | 953.911 | 2153.63 | 519.834 | 993.029 | 1000.27 | 907.835 | 842.189 | 1.49032 | 242.919 |
| Enthalpy Flow | kW | -9.31824 | -41600.3 | -163.981 | -7.27188 | -41780.9 | -768.16 | -42549.1 | -42169 | -41400.9 | -768.098 | -0.0560254 | -41600.3 | -4898.31 | -4424.64 | -3257.82 | -11.9622 | -3269.79 |
| Mass Flows | kg/hr | 96.12 | 9438.26 | 74.218 | 3.4 | 9612 | 240.278 | 9852.28 | 9852.28 | 9612 | 240.278 | 19.2 | 9438.26 | 1205.18 | 1205.28 | 909.555 | 17 | 926.555 |
| BIOMASS | kg/hr | 0 | 0 | 0 | 0 | 0 | 240.278 | 240.278 | 240.278 | 0 | 240.278 | 0 | 0 | 240.278 | 106.59 | 0 | 0 | 0 |
| WATER | kg/hr | 0 | 9438.26 | 0 | 0 | 9438.26 | 0 | 9438.26 | 9438.26 | 9438.26 | 0 | 0 | 9438.26 | 945.7 | 945.7 | 756.56 | 0 | 756.56 |
| NACLO-01 | kg/hr | 96.12 | 0 | 0 | 0 | 96.12 | 0 | 96.12 | 96.12 | 96.12 | 0 | 0 | 0 | 0 | 0 | 0 | 0 | 0 |
| CH3COOH | kg/hr | 0 | 0 | 0 | 3.4 | 3.4 | 0 | 3.4 | 3.4 | 3.4 | 0 | 0 | 0 | 0 | 0 | 0 | 0 | 0 |
| CH3COONA | kg/hr | 0 | 0 | 74.218 | 0 | 74.218 | 0 | 74.218 | 74.218 | 74.218 | 0 | 0 | 0 | 0 | 0 | 0 | 0 | 0 |
| NAOH | kg/hr | 0 | 0 | 0 | 0 | 0 | 0 | 0 | 0 | 0 | 0 | 19.2 | 0 | 19.2 | 19.2 | 19.2 | 0 | 19.2 |
| HCL | kg/hr | 0 | 0 | 0 | 0 | 0 | 0 | 0 | 0 | 0 | 0 | 0 | 0 | 0 | 0 | 0 | 17 | 17 |
| Mass Fractions |  |  |  |  |  |  |  |  |  |  |  |  |  |  |  |  |  |  |
| BIOMASS |  | 0 | 0 | 0 | 0 | 0 | 1 | 0.024388 | 0.024388 | 0 | 1 | 0 | 0 | 0.199371 | 0.0884356 | 0 | 0 | 0 |
| WATER |  | 0 | 1 | 0 | 0 | 0.981925 | 0 | 0.957978 | 0.957978 | 0.981925 | 0 | 0 | 1 | 0.784698 | 0.784628 | 0.831792 | 0 | 0.81653 |
| NACLO-01 |  | 1 | 0 | 0 | 0 | 0.01 | 0 | 0.00975612 | 0.00975612 | 0.01 | 0 | 0 | 0 | 0 | 0 | 0 | 0 | 0 |
| CH3COOH |  | 0 | 0 | 0 | 1 | 0.000353725 | 0 | 0.000345098 | 0.000345098 | 0.000353725 | 0 | 0 | 0 | 0 | 0 | 0 | 0 | 0 |
| CH3COONA |  | 0 | 0 | 1 | 0 | 0.00772139 | 0 | 0.00753308 | 0.00753308 | 0.00772139 | 0 | 0 | 0 | 0 | 0 | 0 | 0 | 0 |

**Table S11: Cellulose extraction stream analysis and properties across designated units**

| **Stream Name** | **Units** | **BBR** | **HCL-1** | **WATER-4** | **S30** | **S20** | **S32** | **S33** | **DISCARD** | **CELLULOSE** |
| --- | --- | --- | --- | --- | --- | --- | --- | --- | --- | --- |
| From |  | F-4 |  |  | M-9 | HX-1 | HEATER-2 | B29 | F-5 | F-5 |
| To |  | HEATER-2 | M-9 | M-9 | HX-1 | HEATER-2 | HX-2 | F-5 |  |  |
| Stream Class |  | MIXCINC | MIXCINC | MIXCINC | MIXCINC | MIXCINC | MIXCINC | MIXCINC | MIXCINC | MIXCINC |
| Temperature | C | 60 | 25 | 25 | 27.3964 | 90 | 100 | 25 | 25 | 25 |
| Pressure | atm | 1 | 1 | 1 | 1 | 1 | 1 | 1 | 1 | 1 |
| Mass Vapor Fraction |  | 0 | 1 | 0 | 0.0120968 | 0.0977917 | 0.982841 | 0.00783861 | 0.00805404 | 0 |
| Mass Liquid Fraction |  | 0.63957 | 0 | 1 | 0.987903 | 0.902208 | 0 | 0.992161 | 0.991946 | 1 |
| Mass Solid Fraction |  | 0.36043 | 0 | 0 | 0 | 0 | 0.0171595 | 0 | 0 | 0 |
| Mass Enthalpy | J/kg | -1.42E+07 | -2.53E+06 | -1.59E+07 | -1.52E+07 | -1.48E+07 | -1.27E+07 | -1.51E+07 | -1.52E+07 | -7.08E+06 |
| Mass Density | kg/cum | 1198.29 | 1.49032 | 993.029 | 107.228 | 8.08081 | 0.613687 | 157.942 | 154.211 | 1173.87 |
| Enthalpy Flow | kW | -1166.82 | -208.282 | -24770.9 | -24979.1 | -24356.4 | -21992.4 | -26040.4 | -25830.5 | -209.824 |
| Mass Flows | kg/hr | 295.73 | 296 | 5620 | 5916 | 5916 | 6211.73 | 6211.81 | 6105.14 | 106.673 |
| BIOMASS | kg/hr | 106.59 | 0 | 0 | 0 | 0 | 106.59 | 0 | 0 | 0 |
| WATER | kg/hr | 189.14 | 0 | 5620 | 5620 | 5620 | 5809.14 | 5809.14 | 5809.14 | 0 |
| CELLULOS | kg/hr | 0 | 0 | 0 | 0 | 0 | 0 | 106.673 | 0 | 106.673 |
| HCL | kg/hr | 0 | 296 | 0 | 296 | 296 | 296 | 296 | 296 | 0 |
| Mass Fractions |  |  |  |  |  |  |  |  |  |  |
| BIOMASS |  | 0.36043 | 0 | 0 | 0 | 0 | 0.0171595 | 0 | 0 | 0 |
| WATER |  | 0.63957 | 0 | 1 | 0.949966 | 0.949966 | 0.935189 | 0.935176 | 0.951516 | 0 |
| CELLULOS |  | 0 | 0 | 0 | 0 | 0 | 0 | 0.0171726 | 0 | 1 |
| HCL |  | 0 | 1 | 0 | 0.0500338 | 0.0500338 | 0.0476518 | 0.0476511 | 0.0484837 | 0 |
